# Real-time colorectal cancer diagnosis using PR-OCT with deep learning

**DOI:** 10.7150/thno.40099

**Published:** 2020-02-03

**Authors:** Yifeng Zeng, Shiqi Xu, William C. Chapman, Shuying Li, Zahra Alipour, Heba Abdelal, Deyali Chatterjee, Matthew Mutch, Quing Zhu

**Affiliations:** 1 Department of Biomedical Engineering, Washington University in St. Louis; 2 Department of Electrical & System Engineering, Washington University in St. Louis; 3 Department of Surgery, Section of Colon and Rectal Surgery, Washington University School of Medicine; 4 Department of Pathology and Immunology, Washington University School of Medicine; 5 Department of Radiology, Washington University School of Medicine

**Keywords:** colorectal cancer, optical coherence tomography (OCT), deep learning, optical biopsy

## Abstract

Prior reports have shown optical coherence tomography (OCT) can differentiate normal colonic mucosa from neoplasia, potentially offering an alternative technique to endoscopic biopsy - the current gold-standard colorectal cancer screening and surveillance modality. To help clinical translation limited by processing the large volume of generated data, we designed a deep learning-based pattern recognition (PR) OCT system that automates image processing and provides accurate diagnosis potentially in real-time.

**Method**: OCT is an emerging imaging technique to obtain 3-dimensional (3D) “optical biopsies” of biological samples with high resolution. We designed a convolutional neural network to capture the structure patterns in human colon OCT images. The network is trained and tested using around 26,000 OCT images acquired from 20 tumor areas, 16 benign areas, and 6 other abnormal areas.

**Results**: The trained network successfully detected patterns that identify normal and neoplastic colorectal tissue. Experimental diagnoses predicted by the PR-OCT system were compared to the known histologic findings and quantitatively evaluated. A sensitivity of 100% and specificity of 99.7% can be reached. Further, the area under the receiver operating characteristic (ROC) curves (AUC) of 0.998 is achieved.

**Conclusions**: Our results demonstrate that PR-OCT can be used to give an accurate real-time computer-aided diagnosis of colonic neoplastic mucosa. Future development of this system as an "optical biopsy" tool to assist doctors in real-time for early mucosal neoplasms screening and treatment evaluation following initial oncologic therapy is planned.

## Introduction

Cancer of the colon and rectum is the second most common malignancy diagnosed globally and represents the 2nd leading cause of cancer mortality worldwide 1. In the US, approximately 145,600 cases of colorectal cancer are diagnosed annually 2. Arising from the inner surface - or mucosal layer - of the colon, these cancers can penetrate through the deeper layers of the colon and spread to other organs. Left untreated, the disease is fatal. Current endoluminal screening or surveillance for colorectal malignancy is performed by flexible endoscopy, which involves visual inspection of the mucosal lining of the colon and rectum with an optical camera mounted on the endoscope. Abnormal appearing areas are then biopsied for histologic analysis. With the current standard of care, there are several shortcomings of endoscopic screening. First, this technique relies on visual detection of abnormal tissue to guide biopsy site selection. However, because small or sessile lesions are hard to detect with the naked eye, early malignancies are often missed 3-5. Second, visual endoscopy can only detect changes in the surface of the bowel wall; while sufficient for screening, this limitation greatly reduces the efficacy of endoscopic surveillance after treatment of certain tumors. In particular, treated rectal tumors can completely disappear from the mucosal surface while still leaving nests of tumor cells hidden beneath the mucosal surface 6-8. To improve screening and surveillance of colorectal cancers, better imaging modalities and methods are needed.

In prior work, several research groups have demonstrated promising results that suggest OCT, an established high resolution imaging modality 9-13, may address the shortcomings of traditional camera endoscopy in the upper gastrointestinal tract 14-16 or large intestine 17-19. OCT has been shown to accurately differentiate abnormal from normal tissue in multiple organs as an “optical biopsy” tool 20-23 in both murine and human colorectal models 24-27. However, clinical application of the technology is complicated by the large volume of data generated and the subtle qualitative differences between normal and abnormal tissue. We hypothesize that computer aided diagnosis (CAD) may be valuable in adapting this modality to clinical applications.

The success of convolutional neural networks (CNN) in computer vision tasks has popularized deep learning for CAD imaging 28-30. CNNs have been applied to OCT images for detecting ophthalmological and cardiac diseases 31-33 as well as segmenting healthy esophagus layers *in vivo* 34. Moreover, CNN has also been applied to colon cancer diagnostics in an image classification style 35-37. Unfortunately, these methods require a large number of labeled training images, making them difficult to develop for clinical applications. Fortunately, recent advances on pattern recognition neural networks make it possible to detect and localize certain objects from a single image 38-40 using a small training dataset. These networks search for multiple patterns in each training image and allow PR-networks to be trained from fewer images as compared with older models. PR-networks have been previously explored in multiple settings 29, however, PR-networks paired with OCT have been unexplored in the colorectal cancer literature.

Here, we report the first study of PR-OCT in differentiating normal from neoplastic colorectal tissue. It is an OCT system trained by RetinaNet, a novel neural network architecture, for pattern recognition tasks. A dentate structural pattern has been utilized as a structural marker of normal specimens and used in PR-OCT prediction. Our method leverages the recent advancement in object detection, which localizes and classifies the diagnostic features at real-time, and achieves an accurate classification result. This initial study demonstrates the feasibility of using PR-OCT as an "optical biopsy" tool to assist doctors in real-time for mucosal neoplasms screening and treatment evaluation following initial oncologic therapy.

## Materials and Methods

### Colon specimen preparation

Patients undergoing extirpative colonic resection at Washington University School of Medicine were recruited prospectively into our study. Immediately following surgical resection, colon specimens underwent imaging of both normal bowel wall as well as areas of known abnormality. For each 3-D imaging task, several volumes of 10 mm x 20 mm x 1.6 mm or 5 mm x 10 mm x 1.6 mm were selected depending on the available time for the image task. Each imaged volume has one 3-D dataset for further data processing. Those scanned volumes were far from each other to preserve independence between data sets. This study was approved by the Institutional Review Board of Washington University School of Medicine, and informed consent was obtained from all patients. All samples were imaged within a one-hour period prior to fixation in formalin for routine pathological evaluation.

### OCT system setup

**Figure 1:**
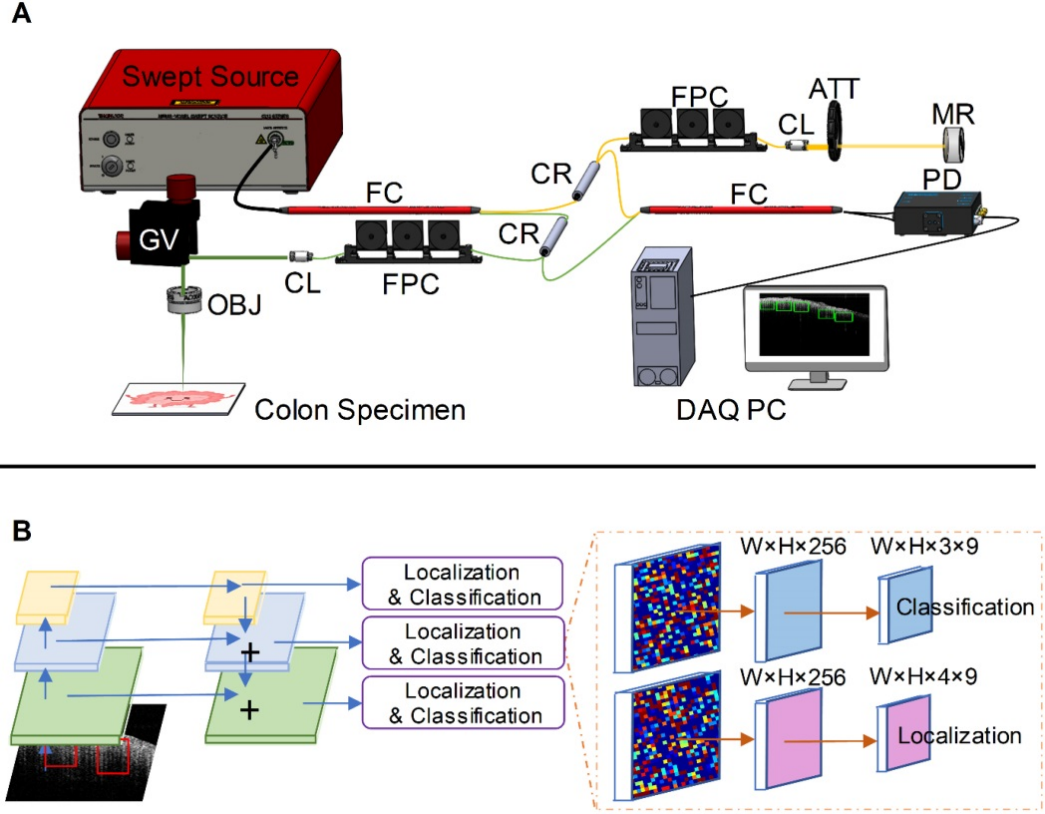
** PR-OCT imaging procedures. A.** Homemade SS-OCT system: FC: fiber coupler, CR: circulator, FPC: fiber polarization controller, CL: collimator, ATT: attenuator, MR: mirror, GV: galvo mirror system, OBJ: objective lens, PD: photodetector, DAQ PC: data acquisition computer; **B.** An illustration of RetinaNet. The left part is an FPN with a ResNet-18 backbone, and the right part are two sub-networks predicting the classifications and locations.

Our swept-source OCT system (Figure 1A) is based on a swept source (HSL-2000, Santec Corp., Japan) with a 1310 nm center wavelength, 110 nm full width at half maximum bandwidth, and 20 kHz scan rate. The input light is split by a 50-50 fiber coupler and then directed to a reference arm and a sample arm by two circulators. Fiber polarization is controlled by two manual fiber polarization controllers. The reference arm is attenuated by a variable density filter. A galvo mirror system (GVS002, Thorlabs) is used to scan the sample arm light beam. The interference signal was detected by a balanced detector (PDB450C, Thorlabs) and sent to a data acquisition board (ATS9462, Alazartec Technologies Inc). Real-time OCT B-scan images are displayed on the monitor. The lateral resolution of the system in air was 10 µm, and the axial resolution was 6 µm by the FWHM definition.

### OCT image labeling and pattern marking

Prior to each imaging study, pathologists or surgical residents provided guidance to the researchers on the sample orientation and location of the examined tumor. Then OCT recorded several datasets accordingly. Each OCT image was labeled as “cancer”, “normal”, “adenomatous polyp”, “treated complete responder”, and “treated non-responder” based on the pathology record of each specimen. This manuscript focuses on identifying normal from malignant specimens, and the preliminary prediction results for other tissue types are also reported.

**Figure 2:**
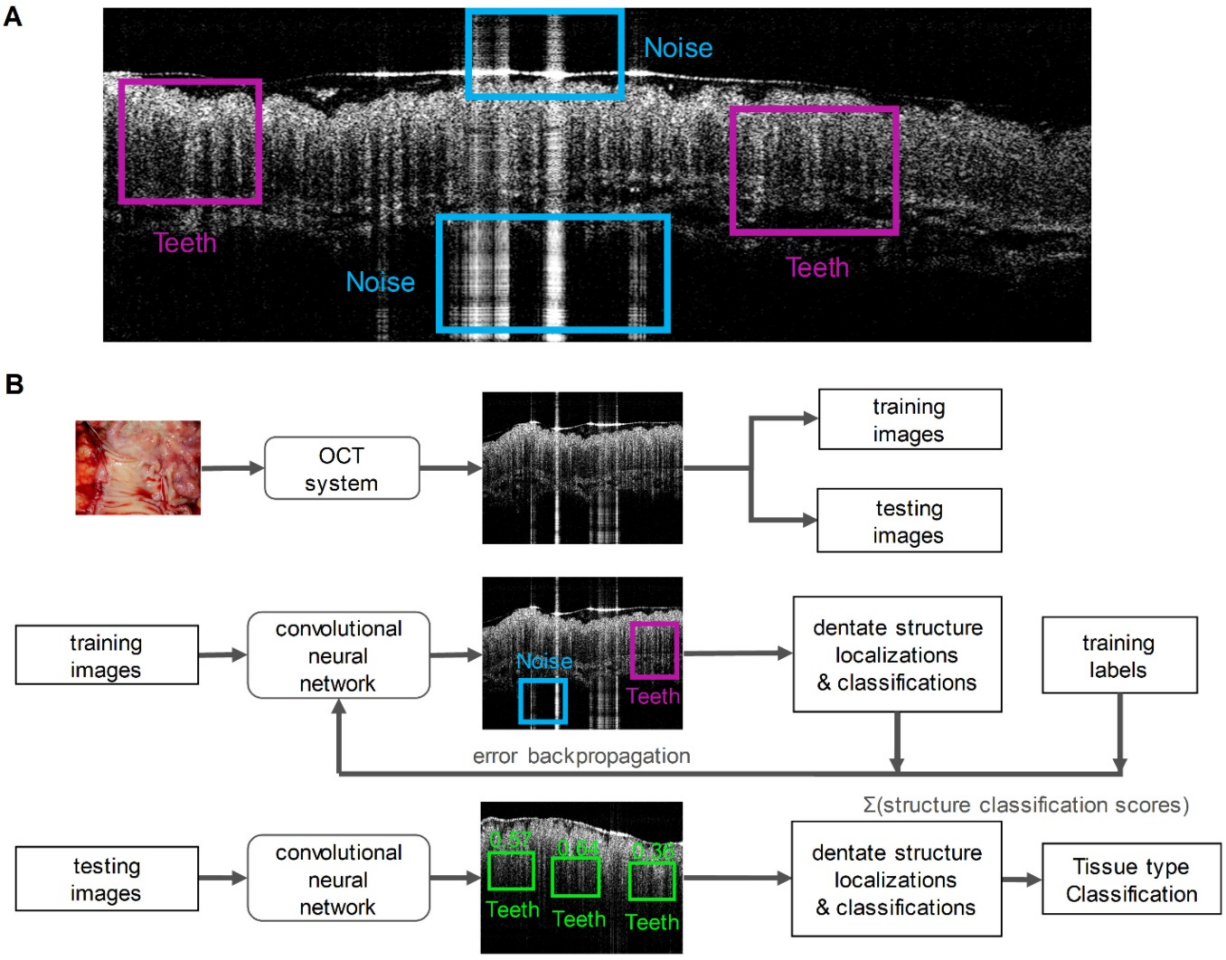
** A.** A training OCT B-scan image from a normal colon. Both “Teeth” and “Noise” classes are labeled with rectangular boxes shown in different colors; **B.** A flowchart summarizes the PR-OCT work flow: first, colorectal B-scan images were collected and separated into training and testing sets; second, “Teeth” and “Noise” patterns were labeled on training images and fed into the RetinaNet; finally, the trained model was tested on all testing images and the performance was evaluated.

Two key imaging patterns were then marked to identify normal colonic mucosa from malignancies: “Teeth” and “Noise”. Literatures previously reported that normal colonic mucosa is associated with a dentate imaging structure, which we termed “Teeth” for this neural network 41,42. The “Noise” category represents strong signals created by hyper-reflection and it has no association with any tissue signature. To train the network, we manually inspected each training B-scan image from both cancer and normal cohorts and marked the specific “Teeth” or “Noise” patterns using the labelImg toolbox. Four researchers were involved for annotating the boxes with a consistent criterion to avoid human bias. Since we detected the features based on the structure rather than the size of the features, we rescaled the input image to a size of 608 × 608, which favors our network structure. The labeled coordinates were also transformed to be registered with the image accordingly. A typical labeled training image is illustrated in Figure 2A.

### Dense object detection with RetinaNet

We used a modified RetinaNet to detect structural patterns associated with normal or malignant tissue 39. The RetinaNet is composed of three parts: a backbone convolutional network that generates feature maps and two subnetworks that perform objection classification and bounding box regression. For our task, we used a feature pyramid network (FPN) backbone on top of a feedforward 18-layer ResNet 43,44. The feature maps generated from the backbone are then fed into a convolutional subnetwork for object classification and boundary coordinates regression, as illustrated in Figure 1B. Four anchors with two aspect ratios ({1:1,1:2}) at two scales are used on each pyramid level 38. Each anchor is assigned with a 3-dimensional one-hot vector representing its class (background, teeth, or noise) and a 4-dimensional vector representing the coordinates of the upper left and lower right corners of the rectangular box that surrounds the objects. The classification is judged by the focal loss 39 and the localization accuracy is evaluated with the robust smooth L1 loss 38. The network is trained for 80 epochs using the Adam solver 45. Though reported successful for many other CAD tasks 29,32, transfer learning techniques are not used here because empirically these methods degrade the performance for our study 46. We suspect this may due to the mismatch between the OCT colon images and the nature photograph images. Thus we train the network from scratch using the labeled OCT images and the Xavier initialization 47.

After training, the model was tested on remaining unseen patients recruited later. During testing, once a pattern was detected in an OCT image, a score was given to estimate the probability of a correct prediction. Then the prediction results were used to classify the image as benign or neoplastic. For each input B-scan image, the RetinaNet provided a list of boxes along with its confidence (probability) belonging to every pattern classes. The score for each B-scan image belonging to the normal class was calculated by summing the “Teeth” confidence value over all the boxes. Finally, we averaged the score over N sequenced OCT B-scan images. This score is used to represent the diagnostic result for the volume corresponding to these N images. Figure 2B summarizes the PR-OCT working flow in a flowchart.

### Statistical analysis

ROC curves were used for the evaluation of our model and the AUCs were used as a performance indicator. With the ground truth acquired from the histology, we categorized the system's prediction as true positive (TP), false positive (FP), true negative (TN), and false negative (FN). We denoted positive as predicting cancer and negative as predicting normal. True and false correspond to the presence of and the absence of a match with the histologic result, respectively. The sensitivity and specificity can then be calculated; from which we plot the ROC by computing the

sensitivity = TP / (TP + FN)

and

1 - specificity = FP / (FP + TN)

using different threshold values for the binary classification. The closer the ROC curve is to the upper left corner, the more accurate the neural network model has performed.

## Results

### Preparation of PR-OCT: establishing OCT dataset and training RetinaNet model

A total of 20 tumor areas, 16 normal areas, 2 adenomatous polyp areas, 2 treated areas from complete responders, and 2 treated areas from non-responders from 24 patients (mean age 69 years old, range: 53-91) were imaged and processed *ex vivo* from August 2017 to July 2019 in Washington University School of Medicine. Diagnoses were ascertained by subsequent surgical pathology examination. Details can be found in Table 1.

In the training cohort of images, 838 labeled OCT images from 4 tumor areas and 4 normal areas acquired from 4 patients were included, where 2176 “Teeth” and 1875 “Noise” patterns were marked. We only used 4 tumor areas and 4 normal areas for training since the AUC of the ROC for our testing set did not improve too much as we included more areas in the training set, as shown in Supplementary Material (Figure S1). The remaining imaged areas, which were not seen by the trained model (from different patients), including 25,250 OCT images were categorized as the testing cohort.

### Qualitative OCT imaging results

**Figure 3:**
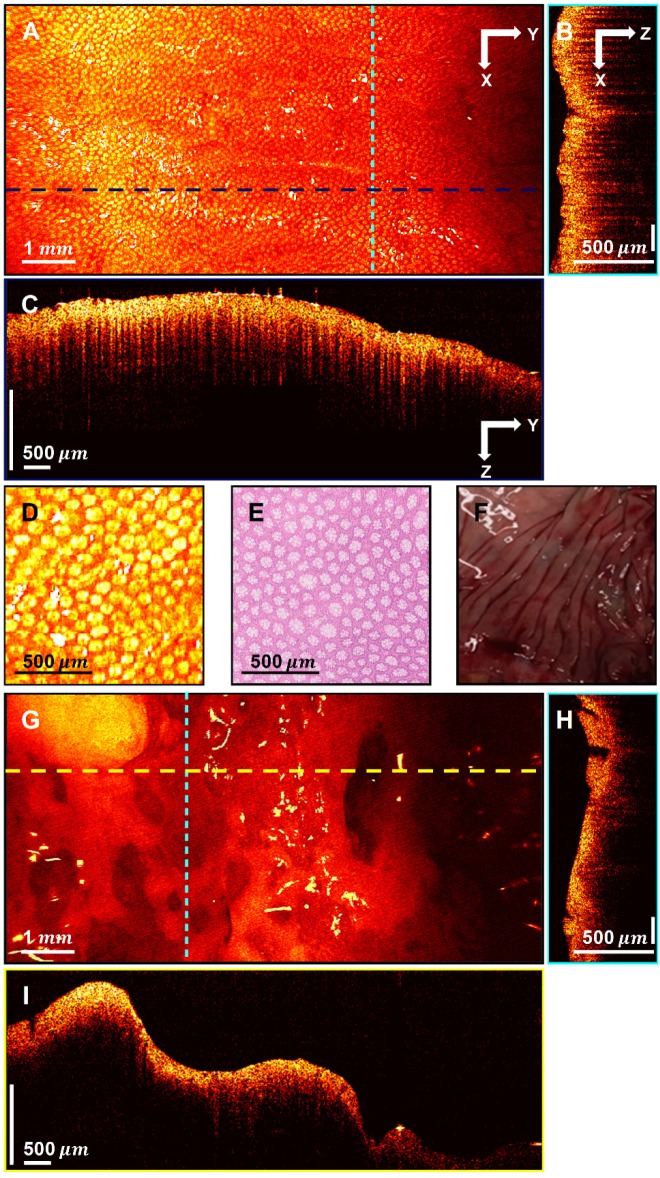
** 3D-OCT images of normal and cancerous human colon specimens. A.** Normal specimen *en face* image constructed by axial summation; **B.** XZ cross-section of normal colon specimen; **C.** YZ cross-section image; **D.** Enlarged area of A; **E.** Representative *en face* histology; **F.** Photograph of a normal specimen; **G.** Cancerous specimen *en face* image constructed by axial summation; **H.** XZ cross-section of cancerous colon specimen; **I.** YZ cross-section image.

Distinct patterns were identified in normal colon tissues. Uniform crypt structures of normal colonic tissue created dentate structures within SS-OCT 3D-scanning images; likewise, the heterogeneous structure distribution of cancerous tissue yielded sparse dentate structures with little organized pattern. Representative images of normal colon tissues, cancerous tissues, and corresponding H&E slides are shown in Figure 3.

Figure 3A displays an *en face* image of a normal colon specimen formed by axial summation along the depth direction (z-dimension) of the entire 3D dataset for visualization. A clear crypt structure can be visualized as dot patterns in the image. When seen in cross-section (Figure 3B in XZ plane and Figure 3C in YZ plane), the uniform crypt structures create a dentate pattern that is replicated throughout normal colonic wall structure. Figure 3D shows an enlarged area in Figure 3A. Figure 3E is a representative *en face* histology image. The OCT and histology images have exactly the same size and come from similar, but not identical, location within the colon specimen. A microstructural *en face* crypt pattern can be clearly visualized in the enlarged area and it correlates well with the histology image. The average crypt diameter is 68 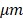 in the enlarged area and 70 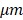 in the *en face* histology, which suggests a close match. Note that tissue fixation as performed on standard pathologic processing results in some tissue shrinkage due to the removal of water from specimens. However, the degree to which this distorts measurements is difficult to assess and occurs more markedly on gross measurements than on microscopic ones. The degree of shrinkage also varies by tissue type; while renal tumors were found to shrink ~10% during fixation process 48, others have found that the majority of shrinkage occurs immediately after resection due to devascularization of the tissue. Since all measurements for this study were taken after resection, this may explain the similarities of size that we found between fresh *ex vivo* measurements and those taken from histology slides after fixation. The photograph of the normal part of the colon specimen is displayed in Figure 3F for reference. Figure 3G shows the *en face* image of a cancerous colon specimen formed by axial summation. There is a heterogeneous structure distribution and the well-organized crypt pattern is broken. This may due to the neoplastic growth. When seen in cross-section (Figure 3H-I), no dentate line can be observed within those cross-sectional images.

### Teeth pattern detection result

**Figure 4:**
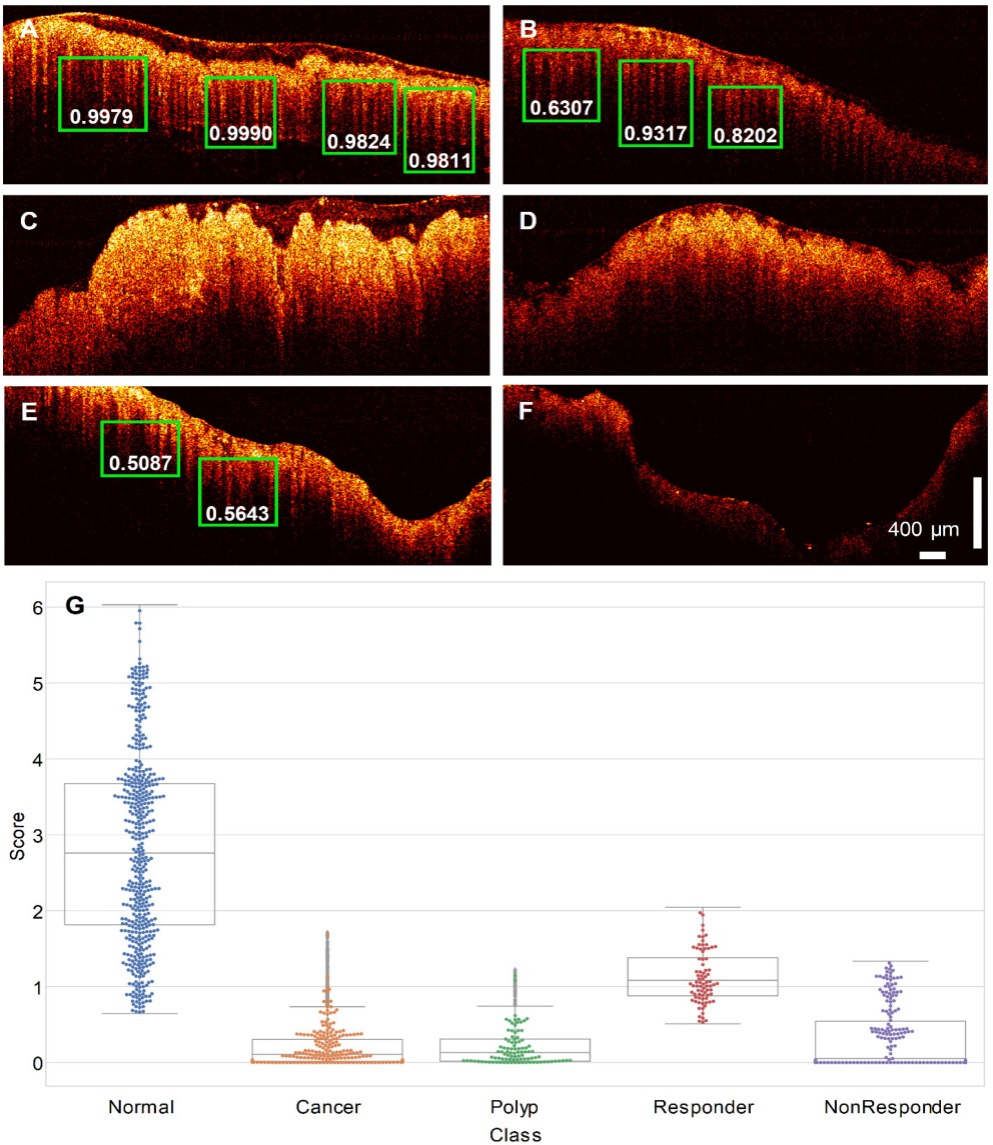
** PR-OCT dentate pattern detection results for: A-B.** normal colon images, green boxes are the predicted “Teeth” patterns and the corresponding scores are labelled on the bottom; **C.** cancer colon images; **D.** polyp colon images; **E.** treated complete responder colon images; **F.** treated non-responder colon images. **G.** A swarm plot on a box plot of prediction scores for normal, cancer, polyp, treated complete responder (Responder in the figure), and treated non-responder (NonResponder in the figure) colon specimens.

The trained RetinaNet was then tested on the testing cohort for pattern recognition purpose. Since the “Teeth” pattern is related to the normality of colon specimens, our network only predicted all “Teeth” patterns within the testing OCT images. Figure 4A-F display pattern recognition results from 6 typical OCT images. In normal cases (Figure 4A-B), the “Teeth” patterns are detected and marked by green boxes with the corresponding scores beside each box. However, no such pattern is detected in the cancerous case (Figure 4C). Figure 4D is the testing result of an adenomatous polyp. No “Teeth” pattern was detected. For the treated complete responders, the “Teeth” patterns come back as shown in Figure 4E. In contrast, no such pattern was detected in treated non-responders (Figure 4F). Only patterns with a score larger than 0.5 are shown for a better visualization. More tested normal and cancer cases can be found in respective Supplementary Video S1, Video S2, Video S3, and Figure S4.

### Identifying colon region with endogenous optical contrast

The identification results of tissue category using the trained neural network are displayed in Figure 4. During testing, N sequenced OCT B-scan images were used for tissue identification. In this report, N was heuristically chosen to be 40, as the AUC of the ROC improves slowly with the increasing of N. Evaluation of different choices of N can be found in Supplementary Material (Figure S2). Figure 4G shows a swarm plot superimposed on a box plot of the prediction scores for the testing cohort. The median value of the normal ones (2.76) is noticeably higher than the cancer ones (0.11). For polyps, the median score is close to cancer (0.13). Treatment responders (median value: 1.08) show a distinct difference to non-responders (median value: 0.05). The treatment responder class has a score closer to normal specimen, and the non-responder class is closer to cancer tissue. The Cohen's d between all scores of five tissue groups can be found in Table 2. Statistically, a larger d means a larger difference between two groups.

**Figure 5:**
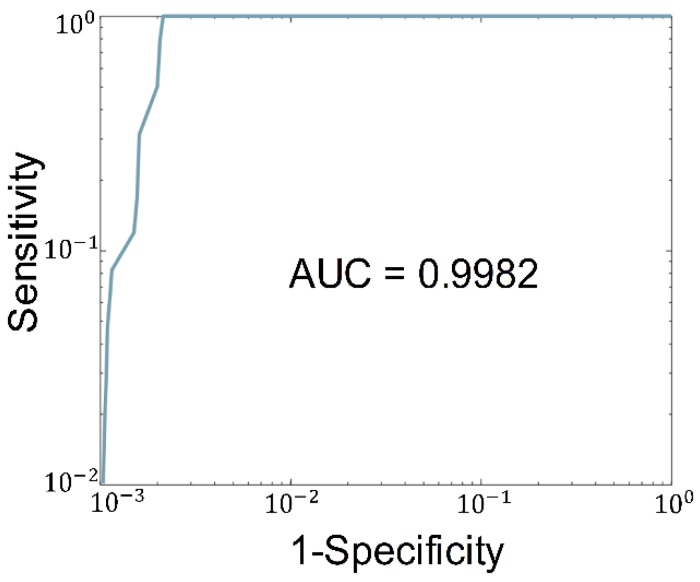
** Plot of the ROC of the binary classification (normal vs. cancer) result.** The AUC is labeled under the ROC.

Figure 5 plots the ROC of the binary classification (normal vs. cancer) result. The true positive rate and the true negative rate are obtained by setting the threshold from 0 to 10. Note that the curve is plotted in the log-log scale because the AUC is very close to 1, which makes the linear scale plot indistinguishable from the boundary. A sensitivity of 100% and specificity of 99.7% can be achieved. The AUC of 0.998 is achieved in our study.

Moreover, we have tested the classification time using different numbers of sequenced OCT images for identification. Using the CPU clock, a total time is calculated by recording the overall time cost to predict 2000 images using a batch size of N on a Nvidia Geforce GTX 1070 GPU. Then we report the classification time as the total time divided by 2000/N. The result can be found in Figure S2. It took around 3.3 s for classifying 40 sequenced OCT images.

## Discussion

This is the first report using a RetinaNet-based PR-OCT system to distinguish normal from neoplastic tissue within human colorectal specimens with real-time diagnosis capability. Using around 26,000 OCT images acquired from 20 tumor areas, 16 normal areas, 2 adenomatous polyp areas, 2 treated areas from complete responders, and 2 treated areas from non-responders, our system has achieved excellent performance. Quantitative scoring of the estimated probability of a normal specimen was used to evaluate performance. The accumulated scores from 40 sequenced OCT images were used for identification of tissue categories and its strength in differentiating cancer and normal in *ex vivo* specimens, with an AUC of 0.998 in 3.3 s.

Previously, a “Teeth” pattern was found as a landmark in OCT images of human normal colon due to the increased optical transmission through the normal crypt lumens 41,42. Consistent results were found in this report. This dentate pattern was therefore used as the basis for tissue type prediction using the RetinaNet system. While we achieve a distinct classification between normal and cancer specimens, the preliminary test on polyps, treated complete responders, and non-responders is also a success. Recent studies have shown that changes in crypt size and appearance are associated with the earliest forms of colorectal cancer 49; therefore, our PR-OCT may lead to more sensitive assessment of early malignancies and improved detection of residual malignant tissue after chemotherapy and radiation treatment.

Clinical translation of PR-OCT requires integration of the probe into the colonoscope for “optical biopsy” in real time during endoscopic evaluation. Several studies have demonstrated the feasibility of endoscopic OCT in both rodent and human models 17-19,26,27, especially, camera-guided endoscopic OCT 24,25. Current screening methods for colorectal tissue rely on histologic evaluation of biopsy specimens, which take days to receive. With a classification time of only few seconds, PR-OCT shows a great potential to provide accurate real-time diagnosis. In addition, PR-OCT was tested on OCT images with different field of views (FoV) and the prediction power was similar. In Supplementary Video S1 and S2, examples with larger FoV can be found; examples of smaller FoV are shown in Supplementary Video S3, and Figure S4. Though we assume the PR-OCT's performance can generalize across different OCT systems, future efforts may focus on testing the performance on other OCT systems, i.e. spectral domain OCT. Therefore, suitable for serving as an “optical biopsy” tool to localize normal and malignant tissues with microscopic resolution. This can help guide more targeted biopsy. Once implemented into colonoscope, it can assist doctors during the colonoscopy procedure to potentially provide a high diagnostic accuracy of early malignancy.

Interestingly, we have achieved an accurate classification on a large amount of unseen testing data set with only limited training images. This is largely due to the training method employed in this study. Rather than throw images along with their classification labels, significant image patterns associate with normal specimens' structure have also provided to the network. In addition, a neural network designed for computer vision tasks is well suited for our objective - detecting one simple “Teeth” pattern in a grayscale image. Therefore, small amount of training data can yield a good prediction result in unseen images. Moreover, the powerful idea of introducing focal loss in RetinaNet dramatically improves the performance of the object detector under the condition of severely unbalanced classes (i.e., thousands of locations are evaluated by the detector, while only a few contain objects). Regarding object detection speed, RetinaNet also gives faster classification than its predecessors because it is a one-stage object detector 38,40,50.

One limitation of the study is the *ex vivo* nature of all imaged specimens. The human *in vivo* environment is likely more complex. For example, bowel movement, surgical adhesions, colonic strictures, etc. can cause difficulties in scanning and imaging. When fully developed, an OCT catheter will be delivered by colonoscope to the area of interest within the bowel 51-54. The system was tested on a very limited number of other abnormalities: 2 adenomatous polyp specimens. As the adenomatous polyp will potentially grow into cancer, it is promising that we got a lower predicting score which was close to cancerous tissue. Additionally, there are other colorectal abnormalities were not tested by PR-OCT, such as inflammatory bowel disease and hyperplastic polyp pathology. The ability to differentiate adenomatous from hyperplastic polyps would make a significant clinical impact. Since most biopsy-proven hyperplastic polyps will not undergo surgical resection due to the nature that they will not grow into malignancy, we did not encounter any patients with incidentally found hyperplastic polyps. We will need to test PR-OCT's ability to differentiate these two types of polyps in future *in vivo* patient studies. Finally, the system was tested on a very limited number of tumors that had previously received radiation and chemotherapy treatment; though the result is promising, the number of specimen is limited. These devised lesions may require more categories in our PR-OCT classification design. It is also worth to mention that if the training sample is too small (i.e. one patient), the prediction power for abnormal lesions will drop as shown in Supplementary Figure S3. Future work includes training the network on an extended training set with more tissue abnormalities from a larger pool of patients.

In conclusion, the results presented suggest that PR-OCT may differentiate normal from cancerous colon rapidly, potentially enabling for real-time use. With further improvement, PR-OCT may enable "optical biopsy" of colorectal tissue in real time, which could direct diagnostic and therapeutic interventions to targeted areas of unusual mucosal growth. While the technology itself is not a direct treatment, one of its potential future applications is to assess the novel “wait and watch” rectal cancer treatment management strategy which allows treatment responders with no residual cancer left to be followed up safely by imaging rather than surgery and therefore preserves their quality of life 55,56. Though promising, these preliminary results warrant further study. Specifically, future efforts will include both hardware and software integration of PR-OCT into the endoscope, fine-tuning the network, and evaluation in the *in vivo* setting.

## Supplementary Material

Supplementary figures.

Supplementary video 1.

Supplementary video 2.

Supplementary video 3.

## Figures and Tables

**Table 1:** Lesion characteristics (patients' mean age 69 years old, range: 53-91)

Pathology reports	Number of imaged areas	Number of OCT images	Average OCT images per area	Median OCT images per area	Average imaged areas per patient	Median imaged areas per patient
**Cancer**	20	12550	628.4	600.0	1.1	1.0
**Normal**	16	8038	502.4	500.0	1.2	1.0
**Adenomatous polyp**	2	2500	1250.0	1250.0	1.0	1.0
**Complete responder**	2	1500	750.0	750.0	1.0	1.0
**Non-responder**	2	1500	750.0	750.0	1.0	1.0

**Table 2:** Cohen's d between all scores of five tissue groups

	Normal	Cancer	Polyp	Responder	Non-responder
**Normal**		3.34	2.47	1.47	2.16
**Cancer**			0.04	3.52	0.43
**Polyp**				3.62	0.44
**Responder**					2.20
**Non-responder**					

## Abbreviations

OCT: optical coherence tomography
PR: pattern recognition
3D: 3-dimensional
ROC: receiver operating characteristic
AUC: area under the curve
CAD: computer aided diagnosis
CNN: convolutional neural networks
TP: true positive
FP: false positive
TN: true negative
FN: false negative
FoV: field of view
