# Supplementary figures and images for: Real-time colorectal cancer diagnosis using PR-OCT with deep learning
Source: Theranostics. 2020 Feb 3;10(6):2587–96. doi: 10.7150/thno.40099 (PMC7052898; doi:10.7150/thno.40099)

## Supplement Figures of Real-time colorectal cancer diagnosis using PR-OCT with deep learning

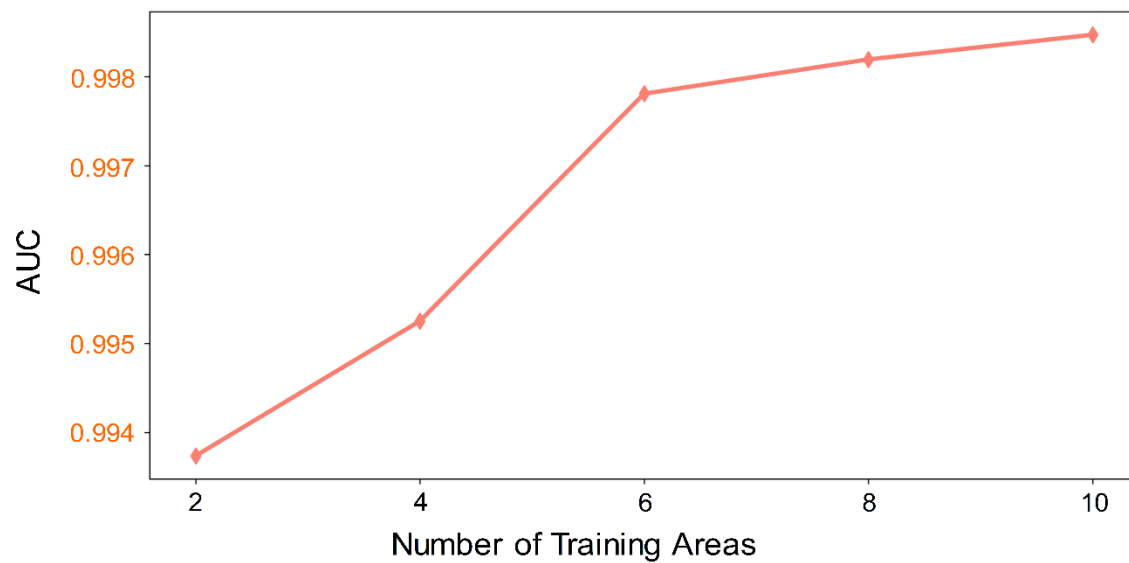

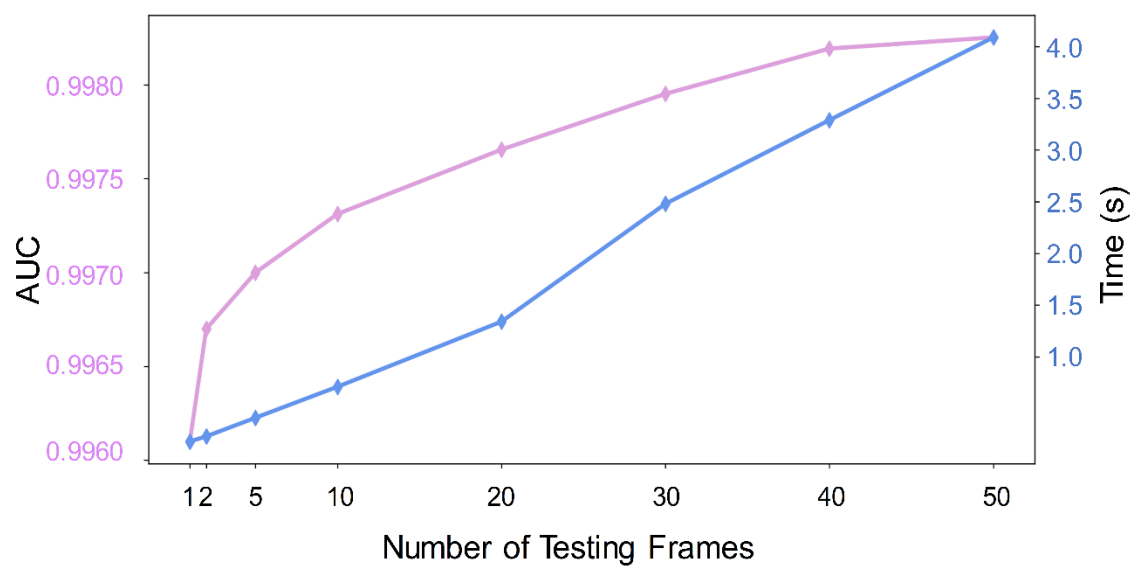

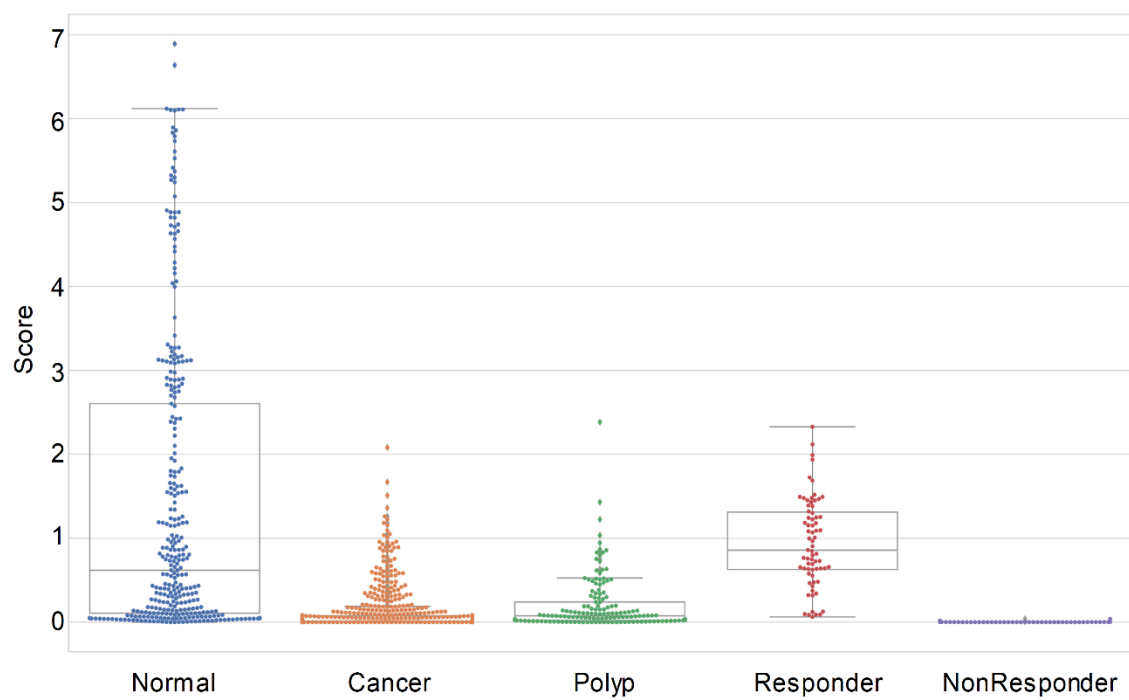

Supplement: Supplementary file 1 — Supplementary figures. [file thnov10p2587s1.pdf]
